# Supplementary material for: Switching to a Standard Chow Diet at Weaning Improves the Effects of Maternal and Postnatal High-Fat and High-Sucrose Diet on Cardiometabolic Health in Adult Male Mouse Offspring
Source: Metabolites. 2022 Jun 18;12(6):563. doi: 10.3390/metabo12060563 (PMC9230726; doi:10.3390/metabo12060563)
Supplement: Supplementary file 1 [file metabolites-12-00563-s001.zip › metabolites-1748066-supplementary.pdf]

Table S1. Summary of qRT-PCR oligonucleotide primers used in measuring mRNA expression of mitochondrial biogenesis, pathological cardiac hypertrophy, interstitial collagen content, and metabolism.

Primer		Sequence
Ppargcα	Forward	GCAGTCGCAACATGCTCAAG
	Reverse	GGGAACCCTTGGGGTCATTT
Tfam	Forward	TCCACAGAACAGCTACCCAA
	Reverse	CCACAGGGCTGCAATTTTCC
Nrf1	Forward	AGAAACGGAAACGGCCTCAT
	Reverse	CATCCAACGTGGCTCTGAGT
Nrf2	Forward	ATGGAGCAAGTTTGGCAGGA
	Reverse	GCTGGGAACAGCGGTAGTAT
Drp1	Forward	ATGCCAGCAAGTCCACAGAA
	Reverse	TGTTCTCGGGCAGACAGTTT
Mfn1	Forward	GCAGACAGCACATGGAGAGA
	Reverse	GATCCGATTCCGAGCTTCCG
Mfn2	Forward	TGCACCGCCATATAGAGGAAG
	Reverse	TCTGCAGTGAACTGGCAATG
Opa1	Forward	ACCTTGCCAGTTTAGCTCCC
	Reverse	TTGGGACCTGCAGTGAAGAA
CypD	Forward	AGATGTCAAATTGGCAGGGGG
	Reverse	TGCGCTTTTCGGTATAGTGCT
Myh6	Forward	ACATTCTTCAGGATTCTCTG
	Reverse	CTCCTTGTCATCAGGCAC
Myh7	Forward	TTCCTTACTTGCTACCCTC
	Reverse	CTTCTCAGACTTCCGCAG
Pln	Forward	GTTGTGCCCTTTTTCTACAC
	Reverse	AGAGAGAGCAGATTTGTGG

Atp2a2	Forward	TGTAAGTGGCCAGATTGCTC
	Reverse	CCTAAACAACTGAAGTTAGG
Acta1	Forward	CGACATCAGGAAGGACCTGTATGCC
	Reverse	AGCCTCGTCGTACTCCTGCTTGG
Nppa	Forward	AGGAGAAGATGCCGGTAGAAGA
	Reverse	GCTTCCTCAGTCTGCTCACTCA
Nppb	Forward	CAGCTCTTGAAGGACCAAGG
	Reverse	AGAGACCCAGGCAGAGTCAG
Col1a1	Forward	GAAACCCGAGGTATGCTTGA
	Reverse	GGGTCCCTCGACTCCTACAT
Col3a1	Forward	CCTGGCTCAAATGGCTCAC
	Reverse	GACCTCGTGTTCCGGGTAT
Col8a1	Forward	CAAGTCCCTCACATGCCTTTG
	Reverse	GCACAGGTGGGATTTCCTTCATA
Ccl2	Forward	GAAGGAATGGGTCCAGACA
	Reverse	ACGGGTCAACTTCACATTCA
Hrpt1	Forward	AGCCCCAAAATGGTTAAGGT
	Reverse	CAAGGGCATATCCAACAACA
B2m	Forward	GTCTTTCTGGTGCTTGTCTC
	Reverse	GTATGTTCGGCTTCCCATTC
Cpt1b	Forward	TTCACTGTGACCCCAGACGGG
	Reverse	AATGGACCAGCCCCATGGAGA
Cpt2	Forward	CTTCTAGAGCCAGAAGTGTTCCA
	Reverse	AGGAGGTGTCTAGCCTTGGTATC
Cd36	Forward	GATGTGGAACCCATAACTGGATTCAC
	Reverse	GGTCCCAGTCTCATTTAGCCACAGT

Glut4	Forward	TCGTCATTGGCATTCTGGTTG
	Reverse	AGCTCGTTCTACTAAGAGCAC
Mcad	Forward	TCGAAAGCGGCTCACAAGCAG
	Reverse	CACCGCAGCTTTCCGGAATGT
Pepck	Forward	CATGACTCGGATGGGCATATC
	Reverse	CATATCCGCTTACAAAGGAGAT
Irs1	Forward	AGCACCTGGTGGCTCTCTACA
	Reverse	CAGCTGCAGAAGAGCCTGGTA
Gapdh	Forward	AGGCCGGTGCTGAGTATGTC
	Reverse	TGCCTGCTTCACCACCTTCT
Actb	Forward	AGAAGCTGTGCTATGTTGCTCTA
	Reverse	TCAGGCAGCTCATAGCTCTTC
36B4	Forward	CTGTGCCAGCTCAGAACACTG
	Reverse	TGATCAGCCCGAAGGAGAAG
Tbp	Forward	GCCTTCCACCTTATGCTCAG
	Reverse	GTTGTTGCTGCTGCTGTTG

**Ppargc1α*, peroxisome proliferator-activated receptor gamma coactivator 1-alpha (PGC1-α); *Tfam*, mitochondrial transcription factor A; *Nrf*, nuclear respiratory factor; *Drp1*, dynamic-related protein 1; *Mfn*, mitofusin; *Opa1*, optic atrophy protein 1; *Myh6*, cardiac α myosin heavy chain; *Myh7*, cardiac β myosin heavy chain; *Pln*, phospholamban; *Atp2a2*, cardiac sarcoplasmic reticulum Ca^{2+} ATPase 2a; *Acta1*, alpha-skeletal muscle actin; *Nppa*, atrial natriuretic peptide; *Nppb*, brain natriuretic peptide; *Col1a1*, collagen type 1 alpha chain; *Col3a1*, collagen type III alpha 1 chain; *Col8a1*, collagen type VIII alpha 1 chain; *Ccl2*, C-C motif chemokine ligand 2; *CypD*, peptidylprolyl isomerase D; *Hprt1*, hypoxanthine phosphoribosyltransferase 1; *B2m*, beta-2-microglbulin; *Cpt*, carnitine palmitoyltransfease; *Cd36*, CD 36 molecule; *Glut4*, glucose transporter type 4; *Mcad*, acyl-coA dehydrogenase medium chain; *Pepck*, mitochondrial phosphoenolpyruvate carboxykinase; *Irs1*, insulin receptor substrate 1; *Gapdh*, glyceraldehyde-3-phosphate dehydrogenase; *Actb*, actin beta; *36B4*, ribosomal protein lateral stalk subunit; *Tbp*, TATA-box binding protein.
